# Comprehensive Analysis of Clinical Trials Registration for Lupus Nephritis Therapy on ClinicalTrials.gov

**DOI:** 10.3389/fmed.2021.680302

**Published:** 2021-06-17

**Authors:** Yanfang Gao, Yuhan Wang, Rongshan Li, Xiaoshuang Zhou

**Affiliations:** ^1^Shanxi Medical University, Taiyuan, China; ^2^Department of Nephrology, The Affiliated People's Hospital of Shanxi Medical University, Shanxi Provincial People's Hospital, Shanxi Kidney Disease Institute, Taiyuan, China

**Keywords:** lupus nephritis, ClinicalTrials.gov, trial registration, drug control, biologics

## Abstract

**Objective:** Clinical trials are the most effective method for evaluating therapeutic strategies. The purpose of this study was to comprehensively assess the characteristics of trials on lupus nephritis (LN) and provide a reference for LN treatment and research.

**Methods:** Registered therapeutic trials on drug interventions for LN were obtained from ClinicalTrials.gov up to December 3, 2020. The general characteristics, methodological characteristics, detailed characteristics, investigated drugs, eligibility criteria, and outcome measures of these trials were analyzed.

**Results:** A total of 126 eligible trials were evaluated, and these trials mainly investigated the initial treatment of adult proliferative LN. Half of the trials enrolled <50 participants, and 70.7% of the trials lasted for 6–24 months. In total, 95.2% of trials adopted an interventional study design. Of intervention trials, 56.6% were in phase 2 or phase 3, 76.7% were randomized, 77.5% employed a parallel assignment, and 41.7% were masked. The eligibility criteria and outcome measures of the included trials varied and involved a variety of indicators. Chemical agents and biologics are the most widely studied immunotherapies, of which mycophenolate mofetil, tacrolimus, and rituximab are the most studied. In addition, some trials studied cell transplantation treatment.

**Conclusions:** The majority of clinical trials for LN therapy registered on ClinicalTrials.gov investigated the initial treatment of adult proliferative LN, and most of these trials were randomized, parallel assigned, and insufficiently masked interventional trials with small scale, short duration, various eligibility criteria, and outcome measures. We hope that more large-scale, long-term multicenter, and high-quality RCT trials with standardized inclusion criteria/exclusion criteria and treatment effect evaluation systems will be conducted and that more energy and funding will be put into exploring biological products and stem cell therapies. In addition, trials for membranous LN, childhood-onset LN, and maintenance phase LN are needed to establish optimal treatment strategies.

## Introduction

Renal involvement is a severe complication of systemic lupus erythematosus (SLE), and biopsy-proven lupus nephritis (LN) occurs in 20–40% of SLE patients (1, 2). LN is typically treated with immunosuppressive drugs, such as glucocorticoids, cyclophosphamide (CTX), or mycophenolate mofetil (MMF). However, these conventional immunosuppressive therapies are not effective for all patients; even in the context of clinical trials, only 50–62% of patients achieve remission. In patients who achieved remission, 18–27% may relapse in 5 years, and 30–37% may relapse in 10 years (3–6). In addition, a considerable proportion (10–30%) of patients will develop chronic renal insufficiency and/or end-stage renal disease (7). Nevertheless, the long-term use of conventional immunosuppressive agents has serious side effects, such as infection risk, bone marrow and gonadal suppression, and bone necrosis. The treatment of LN remains challenging, and more efforts are needed to develop more effective treatment strategies.

In the 1970s, the concept of “clinical trial registration” was proposed in the United States. Clinical trials with positive or promising outcomes are preferred for publication, and clinical trial registration helps to reduce this publication bias (8). ClinicalTrials.gov is a public trial registry provided by the U.S. National Library of Medicine and the U.S. Food and Drug Administration, representing one of the most widely used clinical trial registration platforms worldwide. It has high weekly growth rates for new entries, high transparency and accessibility, and detailed information on past and present clinical trials. Clinical trials are the most effective method to evaluate therapeutic strategies. Evaluating these registered clinical trials will enable us to gain a deeper understanding of the strategy of LN control. Therefore, we searched and analyzed all of these trials on therapy for LN registered in ClinicalTrials.gov to assess the characteristics of registered clinical trials regarding strategy control of LN.

## Materials and Methods

### Data Source

A cross-sectional, descriptive study of clinical trials for LN therapy that had been registered on the ClinicalTrials.gov database was conducted. The trials were obtained from ClinicalTrials.gov using the advanced search function with the search term “lupus nephritis” for “condition” on December 3, 2020. All of these searched clinical trials were assessed to identify records of therapeutic trials. Intervention and observation studies were all included. There were no restrictions on the results of the study, age of the patients, sex, or other enrollment.

### Statistical Analysis

We extracted all the following information: National Clinical Trial (NCT) number, study type, study start date, during date, enrollment, participant age, gender, locations, recruitment status, study results, publications of the study, sponsor, collaborators, funding type, Data Monitoring Committee (DMC), U.S. FDA-regulated product, Individual Participant Data (IPD) sharing statement, phases, allocation, intervention model, masking, primary purpose, groups or arms, time perspective, observational model, experimental medications, eligibility criteria, and outcome measures. The general characteristics of the clinical trials are presented as descriptive statistics. Categorical data were expressed by calculating the frequency and percentage. All analyses were performed using Microsoft Excel software.

## Results

### General Characteristics of the Included Clinical Trials

**Figure 1:**
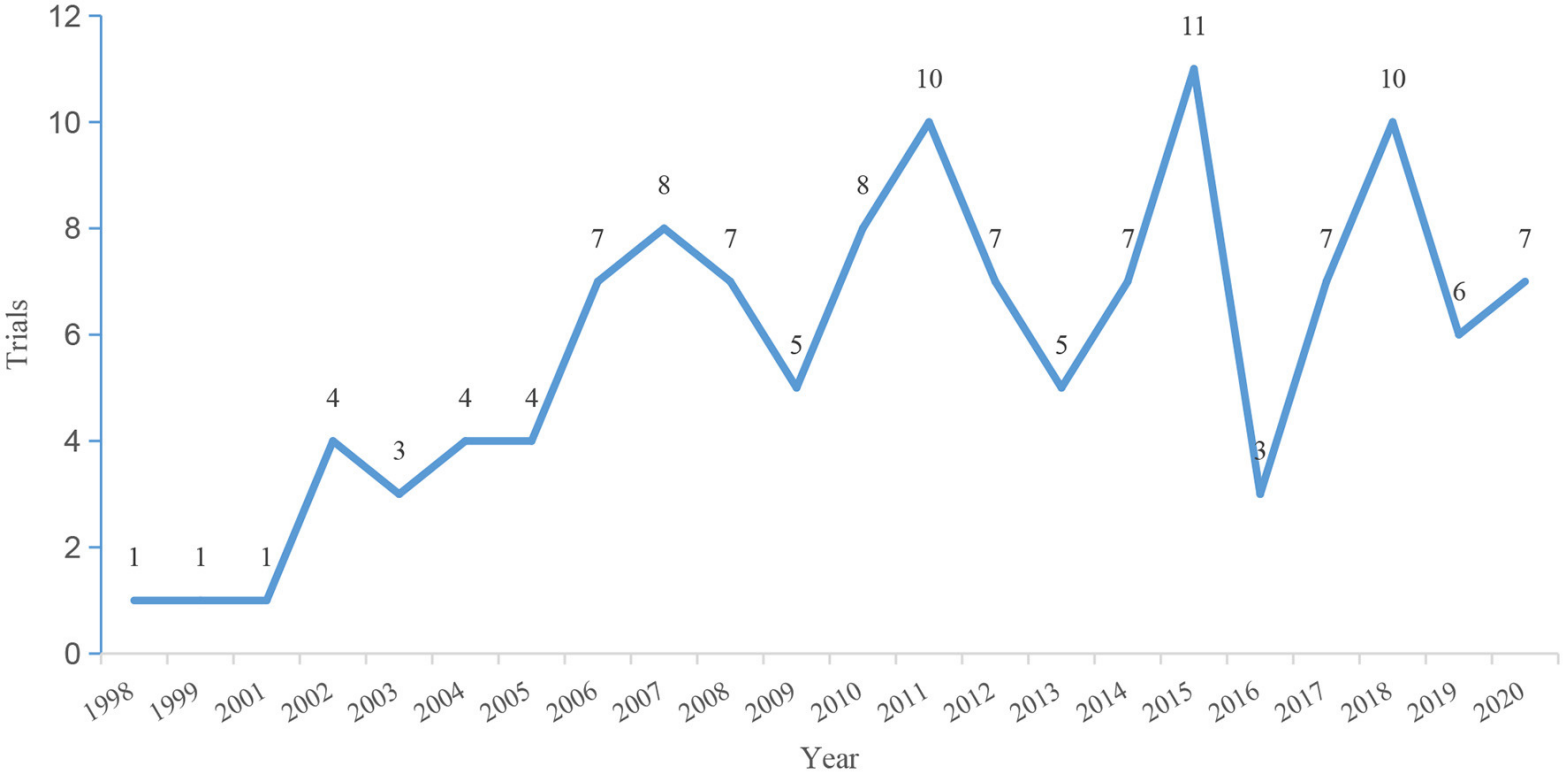
Quantity trend of registered trials per year.

**Figure 2:**
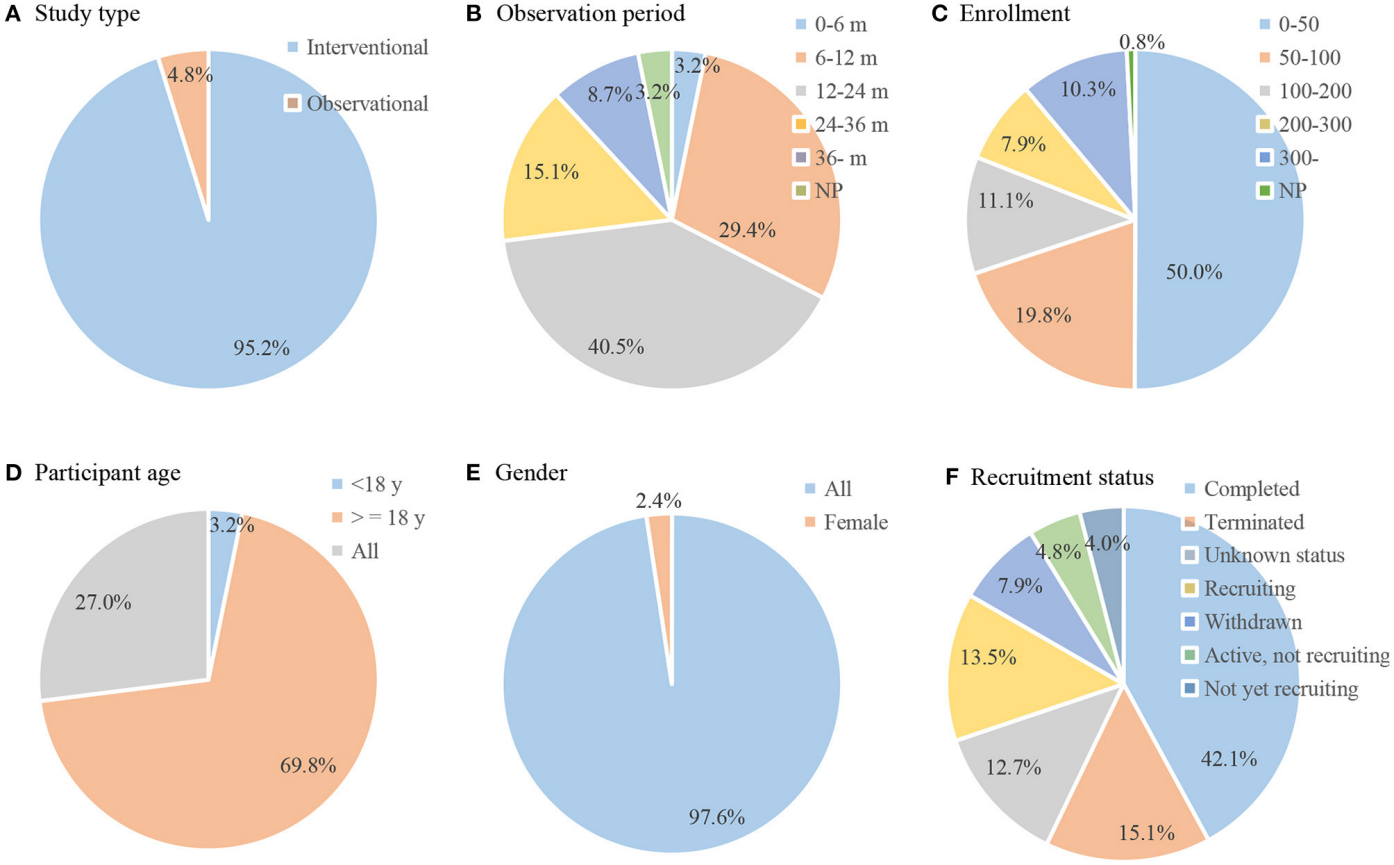
General characteristic of included trials (*n* = 126). Pie chart of **(A)** study type; **(B)** observation period; **(C)** enrollment; **(D)** participant age; **(E)** gender; **(F)** recruitment status.

The initial search identified 159 clinical trials for LN therapy registered on ClinicalTrials.gov up to December 3, 2020. After carefully reviewing all the information, 33 trials were not therapeutic trials in patients with LN and were excluded. Thus, a total of 126 registered trials were included. The enrolled trials were registered between 1998 and 2020. The number of registered trials has increased significantly since 2006, and most trials (*n* = 108, 85.7%) began in 2006 and after (Figure 1). On average, there are approximately seven trials every year. The general characteristics of the identified trials are shown in Figure 2. Of the eligible trials, 120 (95.2%) were interventions, and only 6 (4.8%) trials were observational trials. Of all trials, 29.4% spanned 6–12 months, 40.5% spanned 12–24 months, and 15.1% spanned 24–36 months. Only 8.7% trials were conducted for 36 months or more. Only five trials lasted 5–10 years. Half of these trials enrolled <50 participants (*n* = 63, 50.0%). Only 29.3% (*n* = 37) recruited 100 or more participants, 18.2% (*n* = 23) recruited 200 or more participants, and 10.3% (*n* = 13) recruited 300 or more participants. Most trials (*n* = 88, 69.8%) included adults only. Some trials (*n* = 34, 27.0%) included both children and adults, and a small number of trials (*n* = 4, 3.2%) exclusively included children. Almost all subjects (*n* = 123, 97.6%) in these trials did not restrict the gender of the participants. Only three studies included only women. Completed status was dominant in the included trials (*n* = 53, 42.1%). Nineteen trials (15.1%) were terminated, and 10 trials (7.9%) were withdrawn. Insufficient enrollment (*n* = 10), safety concerns (*n* = 4), and insufficient efficacy to warrant continuation of the study (*n* = 5) were the main reasons. Other reasons included funding problems (*n* = 1), administrative reasons (*n* = 1), and implementation issues (local pharmacies unwilling to comply with the study protocol (*n* = 1).

### Methodological Quality of Included Clinical Trials

**Figure 3:**
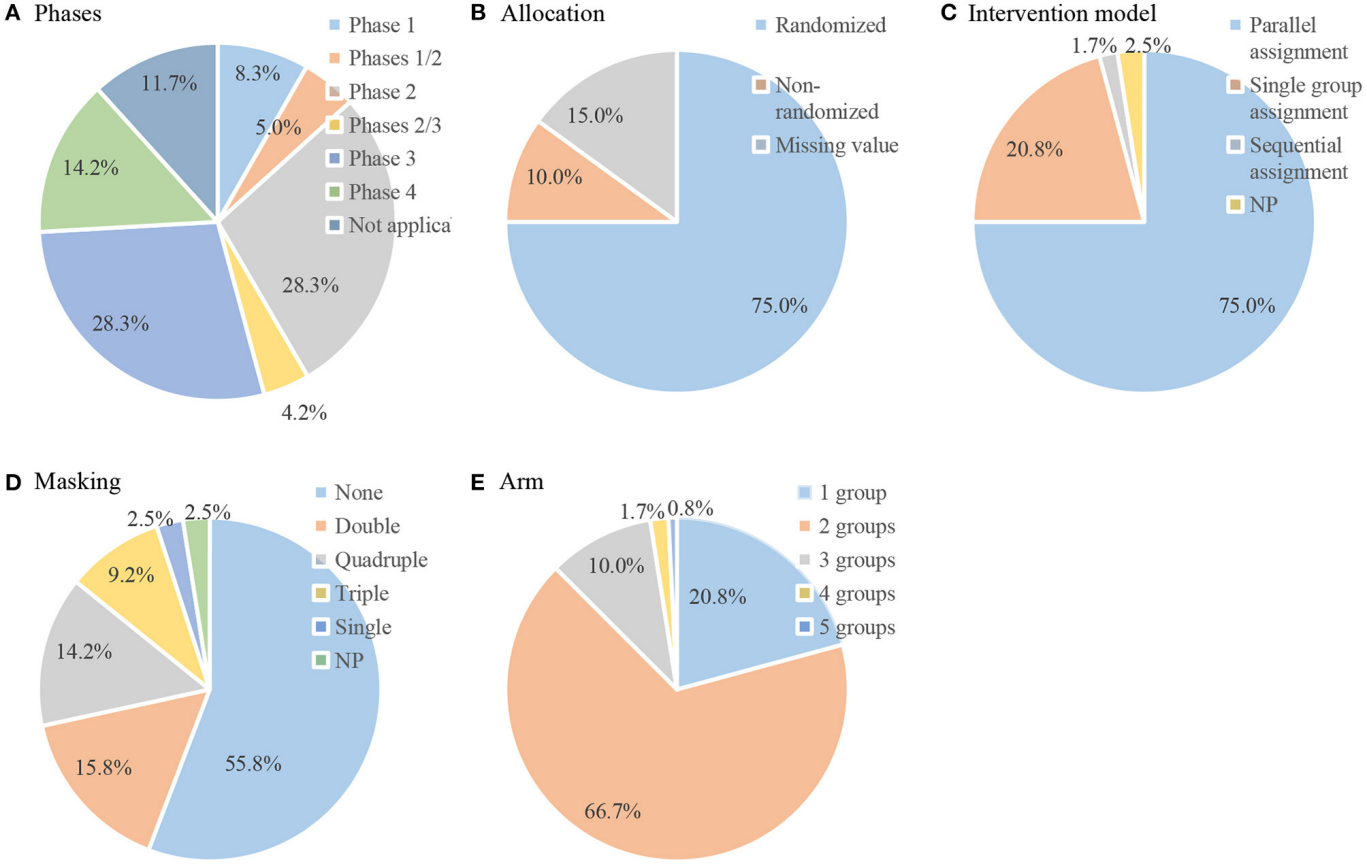
Design data of included interventional trials (*n* = 120). Pie chart of **(A)** phases; **(B)** allocation; **(C)** intervention model; **(D)** masking; **(E)** arm.

The study design characteristics of the included trials are displayed in Figure 3. Among the 120 interventional trials, most trials (*n* = 73, 60.8%) were in phase 2 or 3. Of the interventional trials, 75.0% were randomized, 75.0% were parallel assignment, and 55.8% were not masked. A total of 79.2% of the trials were divided into two or more groups. In addition, of the six observational trials, half were cohort studies, and half were case-only studies. Five of the trials were prospective.

### Detailed Characteristics of the Included Clinical Trials

**Figure 4:**
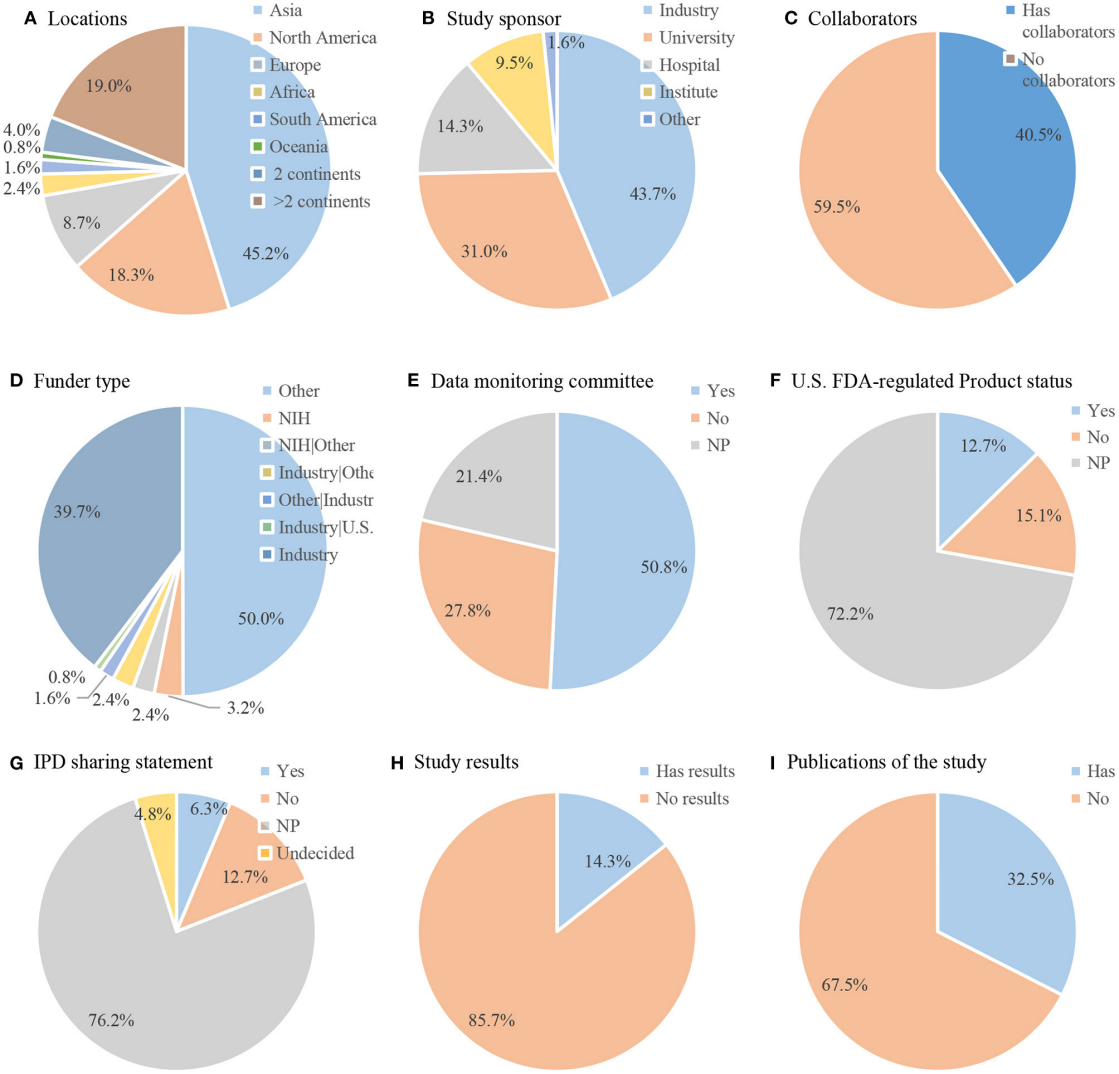
Detailed characteristics of included trials (*n* = 126). Pie chart of **(A)** locations; **(B)** study sponsor; **(C)** collaborators; **(D)** funder type; **(E)** data monitoring committee; **(F)** U.S. FDA-regulated Product status; **(G)** IPD sharing statement; **(H)** Study results; **(I)** Publications of the study. IPD, Individual Participant Data.

Detailed characteristics of the included trials are displayed in Figure 4. The selected 126 trials were implemented on six continents. Most (*n* = 97, 77.0%) were conducted on only one continent, mainly in Asia (*n* = 57, 45.2%), North America (*n* = 23, 18.3%), and Europe (*n* = 11, 8%). Twenty-nine (23.0%) trials were conducted on two or more continents, of which 5 trials (4.0%) were conducted on two continents and 24 trials (19.0%) were conducted on more than two continents. Industries were listed as primary sponsors in 43.7% trials, universities in 31.0% trials, and hospitals in 14.3% trials. Greater than one-third (*n* = 40, 40.5%) of trials involved collaborations. Most of the trials (*n* = 63, 50.0%) were supported by other types of funds followed by industrial funds (*n* = 50, 39.7%). Only 4 (3.2%) trials were funded by the NIH. Half of the trials (*n* = 64, 50.8%) provided DMC. A total of 12.7% of the treatment regimens used were US FDA-regulated products. In total, 6.3% had IPD sharing statements. Eighteen (14.3%) trials listed results on ClinicalTrials.gov, whereas 41 (32.5%) trials had web links for the publications of results on ClinicalTrials.gov.

### Overview of Investigated Strategy

An overview of the treatment strategy for LN in the registered clinical trials is shown in Table 1. The majority of the trials investigated the initial treatment of LN, and the drugs involved in the trials were divided into three categories: chemical agents, biologics, and cell transplantation. Among them, conventional chemical agents were the most studied (*n* = 67) followed by biological agents (*n* = 41). In addition, 11 trials investigated the effect of stem cell transplantation. Among the chemical agents, the most studied were calcineurin inhibitors (CNIs) followed by antiproliferative/antimetabolite agents. CNIs includes tacrolimus (TAC), cyclosporine A (CsA), and voclosporin. Twenty-nine trials investigated the efficacy and safety of CNIs. Among them, 20 trials investigated CNIs only, and 9 trials investigated CNIs combined with MMF. TAC was the most investigated drug (*n* = 22). The most investigated antiproliferative/antimetabolite agents are CTX (*n* = 9) and MMF/mycophenolic acid (MPA) (*n* = 8). In addition, azathioprine, leflunomide, sirolimus, mizoribine, deoxyspergualin, and fludarabine were also investigated. Other chemical agents, including proteasome inhibitors (ixazomib, velcade, KZR-616), immunomodulators (*Tripterygium wilfordii* and laquinimod), antimalarial drugs (chloroquine and artesunate), and others (acthar gel calcitriol, iguratimod, pentoxifylline, tamibarotene), were also investigated. Twenty-seven types of biologics were investigated, and the most studied single drug in biologics was rituximab (*n* = 7). Among the cell transplantation treatments, bone marrow-derived mesenchymal stem cells (*n* = 4) and umbilical cord-derived mesenchymal stromal cells (*n* = 4) were the most studied. Of the eligible trials, only 13 trials investigated maintenance therapy and involved 10 types of drugs, such as MMF, abetimus sodium, BI 655064, prednisolone, TAC + MMF, TAC, leflunomide, MMF/azathioprine, and *Tripterygium wilfordii*.

**Table 1:** Overview of investigated strategy.

**Types**		**Name of drugs**	**Number**
Chemical agents			67
	Antiproliferative/ Antimetabolite agents		
		MMF/MPA	8
		CTX	9
		Azathioprine	1
		Leflunomide	1
		Sirolimus	1
		Mizoribine	1
		Deoxyspergualin	1
		Fludarabine	1
	Calcineurin inhibitors		
		TAC	18
		Cyclosporin	2
		TAC+MMF	4
		Voclosporin+MMF	4
		Cyclosporine+MMF	1
	Proteasome inhibitors		
		Ixazomib	1
		Velcade	1
		KZR-616	1
	Immunomodulators		
		*Tripterygium wilfordii*	1
		Laquinimod	1
	Antimalarial drugs		
		Chloroquine	1
		Artesunate	1
	Other		
		Acthar gel	2
		Calcitriol	2
		Iguratimod	2
		Pentoxifylline	1
		Tamibarotene	1
Biological agents			41
	T-cell directed		
		Abatacept	4
		RG2077	1
		BI 655064	1
		Iscalimab	1
		BG9588	1
		Itolizumab	1
		Milatuzumab	1
	B-cell directed		
		Rituximab	7
		Ocrelizumab	1
		Obinutuzumab	2
		Atacicept	2
		Blisibimod	2
		Belimumab	2
		Belimumab + Rituximab + Cyclophosphamide	1
	Cytokine directed		
		BIIB023	2
		Infliximab	1
		AMG 811	1
		Anifrolumab	1
		Etanercept	1
		CNTO 136	1
		Secukinumab	1
		Guselkumab	1
		Anti-MIF antibody	1
	Targeting complement components and signal path passage		
		Ravulizumab	1
		APL-2	1
		Narsoplimab	1
		BMS-986165	1
	Stem cell transplantation		11
		Bone marrow derived mesenchymal stem cells	5
		Umbilical cord-derived mesenchymal stromal cells	4
		Amniotic mesenchymal stem cell	1
		Hematopoietic stem cell	1
Maintenance therapy			13
		MMF	4
		Abetimus sodium (LJP 394)	2
		BI 655064	1
		Prednisolone	1
		TAC+MMF	1
		TAC	1
		Leflunomide	1
		MMF/Azathioprine	1
		Tripterygium wilfordii	1

### Eligibility Criteria of Included Clinical Trials

The inclusion/exclusion criteria of the included trials varied and involved a variety of indicators, such as pathological type, proteinuria level, serum creatinine, estimated glomerular filtration rate (eGFR), systemic lupus erythematosus disease activity index (SLEDAI), albumin level, urinary sediment, and anti-dsDNA antibody level. The eligibility criteria of each indicator also differed. We compared and analyzed four important and frequently used indicators of the inclusion/exclusion criteria of the included trials, and the results are shown in Table 2. The inclusion/exclusion criteria of 83 trials specified the pathological types of LN patients. Most of the trials included the pathological type of proliferative LN (*n* = 43) or mixed proliferative LN and membranous LN (MLN) (*n* = 34). Only six trials included pure MLN. Among the included trials, 79 trials specified the proteinuria level (urine protein to creatinine ratio (UPCR) or 24-h urinary protein excretion level) of LN patients. Nine trials (five included pure MLN and four included mixed membranous and proliferative LN) defined the proteinuria level of MLN patients. The level was >2 g/g in four trials, >3 g/g in four trials, and >3.5 g/g in one trial. Among the other 74 trials, 37 trials included LN patients with proteinuria >1 g/g, 14 trials included patients with proteinuria >0.5 g/g, 6 trials included patients with proteinuria >1.5 g/g, and 5 trials included patients with proteinuria >2 g/g. The eligibility criteria of 42 trials had provisions for eGFR, and 26 patients with eGFR >30 ml/min/1.73 m^2^ were included. One-third of the trials involved serum creatinine levels, but the eligibility criteria of each trial differed. Only 18 trials had SLEDAI scores in the eligibility criteria, and most trials had SLEDAI scores >8 or 10.

**Table 2:** Eligibility criteria of included clinical trials.

**Eligibility criteria**			**Number**
Pathological types			
	Proliferative LN		43
		III/IV	23
		III/IV/III+V/IV+V	16
		II/III/IV	2
		IV/IV+V	1
		II/IV	1
	Proliferative LN and membranous LN		34
		III/IV/V	17
		III/IV/V/III+V/IV+V	12
		IV/III+V/IV+V/V	2
		II/III/IV/V	1
		II+V/V	1
		III+V/IV+V/V	1
	Pure membranous LN	V	6
Proteinuria level (g/g)			
	V		9
		>2	4
		>3	4
		>3.5	1
	Other		74
		>0.15	1
		>0.5	14
		>0.75	1
		>1	37
		>1.5	6
		>2	5
		>3	1
		>3.5	1
		>1/1.5	1
		>1/2	3
		<1	3
		<3.5	1
Creatinine (mg/dl)			41
		<0.3	2
		<1.2	1
		<1.5	3
		<1.6	1
		<2	4
		<2.3	3
		<2.5	4
		<2.8	1
		<3	12
		<3.4	2
		<5	1
		>1	1
		>1.3	1
		>1.3 and <4	3
		>1.5	1
		>1.8	1
eGFR (ml/min/1.73 m^2^)			42
		>15	1
		>15 and <60	1
		>20	5
		>30	27
		>35	1
		>40	2
		>45	2
		>50	2
		>60	1
SLEDAI			18
		>4	1
		>6	2
		>8	7
		>10	4
		>12	2
		<10	1
		<4	1

### Outcome Measures of Included Clinical Trials

The included trials involved a wide variety of primary and secondary outcome measures. The top 20 primary and secondary outcome measures are summarized in Table 3. Of the composite outcomes, complete remission (CR), total remission (TR), partial remission (PR), and flare are used most frequently followed by changes from baseline in UPCR, eGFR, anti-dsDNA antibody level, complement 3 (C3) level, and complement 4 (C4) level, which are used to evaluate urine protein, renal function, and lupus serological activity, respectively. There were 48 trials using CR as the outcome. Among these trials, 38 trials specified the definition of CR, and there were 37 different definitions. PR was used as the outcome in 30 trials. Among these trials, 29 trials specified the definition of PR, and there were 24 different definitions. The general standard of TR is CR+PR, and nine tests specified other definitions of TR. CR and PR standards generally consist of two or more of the following indicators: UPCR, 24-h urine protein, eGFR, serum creatinine, serum albumin, and urine sediment. Regarding the definition of CR, UPCR+eGFR or UPCR+ serum creatinine with or without urinary sediment is the most common. With regard to the definition of PR, UPCR is defined alone, and UPCR+ serum creatinine is the most common.

**Table 3:** The top 20 primary and secondary outcome measures.

**Outcome measures**	**Number**	**Outcome measures**	**Number**
CR	48	Serum creatinine level	13
TR	47	SLEDAI score	12
PR	30	Renal flare	12
Change from baseline in UPCR	17	Extra-renal flare	11
Flare	16	Change from baseline in serum creatinine level	11
Change from baseline in anti-DNA antibody	15	Change from baseline in SLEDAI	10
Change from baseline in C3	14	Change from baseline in serum albumin	10
Change from baseline in eGFR	14	C3 level	9
Change from baseline in C4	13	Anti-dsDNA antibody level	9
Time to CR	13	Treatment failure	8

Half of the studies evaluated the safety and tolerability of the treatment strategy. Safety and tolerability assessments included clinical manifestations, physical examination, vital signs, laboratory tests, laboratory tests (including hematology, serum chemistry, and urinalysis), side effects, and adverse events (AEs). AEs were assessed by the common terminology criteria for adverse events (CTCAE) v4.0 or v5.0 and relevant evaluation indicators, including the number and percentage of patients with AEs, serious adverse events (SAEs), AE leading to study discontinuation, and AE of special interest (infusion-related reactions, neutropenia, infections, thrombocytopenia, gastrointestinal symptoms, transient increases in serum creatinine, liver function disorder and glucose intolerance, hospitalization, death, etc.).

## Discussion

This study comprehensively analyzed therapeutic trials in patients with LN registered on ClinicalTrials.gov. Through this analysis, we found that the majority of clinical trials for LN therapy registered on ClinicalTrials.gov were randomized, parallel assigned, and insufficiently masked interventional trials with a limited number of participants, short duration, and various eligibility criteria and outcome measures. Chemical agents and biologics are the most widely studied immunotherapies, of which MMF, TAC, and rituximab are the most studied. Most trials investigated the initial treatment of adult proliferative LNs, and only a few trials included children, membrane LNs, and maintenance treatment phase LNs. Very few completed trials have results available on ClinicalTrials.gov, and few trials have IPD sharing statements.

From the perspective of study design, the majority of the trials clearly indicated random allocation (75.0%) and parallel assignment models (75.0%). Only 41.7% trials indicated masking procedures. The essential elements of designing a worthwhile clinal trial include ensuring that the trial provides an unbiased treatment comparison by appropriate randomization, blinding, and making the trial large enough so that it is adequately powered to detect (or refute) any treatment differences of clinical importance (9). The application of randomization can essentially eliminate the influence of reverse causality and selection bias on research validity and significantly mitigate the influence of confusion (10). In the double-blind method, patients and the evaluators responsible for their treatment and follow-up are not aware of which randomized treatment is assigned to patients to avoid any potential impact treatment awareness on patients' cognition, patient management, and endpoint evaluation (11, 12). According to these registrations, the scale of most studies was still small. Half of these trials included <50 patients, and only 29.3% recruited >100 participants. Large-scale multicenter trials facilitate the recruitment of a sufficient number of patients with rapid trial progression compared with single-center trials (13). For chronic disease trials, sample scale determination involves considering the length of follow-up as well as patient numbers (9). LN is a lifelong autoimmune disease. Patients who achieve remission may relapse in the future, and some patients will develop chronic renal insufficiency and/or end-stage renal disease. Reasonable evaluation of treatment strategies should include short-term therapeutic effects and long-term prognosis. Of the included trials, 32.6% spanned <12 months, and only 8.7% spanned more than 36 months. In view of proteinuria at 12 months representing the best single predictor for long-term renal outcome (i.e., risk for end-stage kidney disease or doubling of serum creatine levels after 10 years) (14–16) and limited practical conditions, the duration of the study should be at least 12 months. Based on the above, we hope that more large-scale, long-term multicenter and sufficiently masked high-quality RCT trials will be conducted henceforth.

The inclusion/exclusion criteria of the included trials varied. The inclusion/exclusion criteria involved a variety of indicators, and each indicator differed. LN treatment effect evaluation includes many aspects: renal disease activity (proteinuria, renal function, urinary sediment, albumin, prednisone dose, and renal biopsy), overall disease activity, quality of life, immunological activity (autoantibodies, complement, immune cells, and cytokines), and other outcomes (survival rate and death). Each aspect has a variety of evaluation indicators. Each evaluation indicator can include a variety of different outcome indicators. For example, UPCR can have multiple outcomes: UPCR level, change from baseline in UPCR, number of patients with UPCR <0.75 g/g, and number of patients with a reduction from baseline in UPCR by at least 50%. Moreover, some indicators have a variety of measurement methods (e.g., UPCR can be measured over a 24-h urine collection, a single random urine collection, or a morning first urine collection) and calculation methods (e.g., eGFR can be calculated using a variety of formulas). In addition, different laboratories have different reference ranges and different detection time points. Therefore, we can see that the outcome measures of the trials vary.

Composite outcomes can comprehensively reflect the degree of disease through the combination of multiple outcome indicators. CR, PR, and TR were the most frequently used composite outcomes. At present, there is no unified definition for CR, PR, and TR of LN. Through the analysis of the outcome measures of the included trials, many problems in the application of composite outcomes were identified. (1) Some trials did not specify the definition of the composite outcome. Forty-eight trials applied CR as an outcome, but 11 of them did not have a specified definition of CR. (2) Some definitions are unclear, such as normalization of serum creatinine, inactive urinary sediment, return to normal, and return to baseline. (3) The definitions of the composite outcome are different. Thirty-eight trials defined CR, but only two of the definitions were consistent. Analyzing the differences among the definitions, the main differences were identified. On the one hand, the number and types of indicators included in the composite outcome differ. On the other hand, the criteria of the included indicators differ, including different contents (whether the urine sediment includes white blood cells), different reference objects (normal value or baseline value or both), different cutoff point values, and whether the cutoff point value is included. Finally, some definitions require additional conditions (e.g., six CR and four PR required prednisone dose tapered to <10 mg/day). Unclear definitions and different definitions made the same composite outcome represent different outcomes. In summary, the inclusion/exclusion criteria and outcome measures of the included trials varied, which resulted in a lack of uniformity and comparability with similar studies. It is necessary to standardize the inclusion/exclusion criteria and treatment effect evaluation system of general LN clinical trials to improve the quality of the research, increase industry comparability (external validity), and provide effective evidence for evidence-based medicine.

Currently, proliferative LNs, including type III, type IV, type III plus V, and type IV plus V LNs, are severe clinical entities with poor prognosis. Both MMF/MPA and low-dose CTX are recommended as first-line options for initial treatment (17–19). Despite improvements in kidney failure-free survival, a considerable number of patients do not achieve remission with the recommended induction regimens, and more efforts are needed to develop more effective treatment strategies. Focus has been placed on the use of CNIs. The mechanism of CNIs in treating LN includes immune modulatory effects and nonimmune-mediated mechanisms. CNIs inhibit calcium- or calmodulin-dependent phosphatase, which leads to a decrease in the nuclear translocation of transcription factors related to interleukin-2 transcription (such as NF-AT), thus blocking T-cell activation (20). Nonimmune-mediated mechanisms include CNIs enhancing podocyte function by stabilizing the actin cytoskeleton, improving podocyte survival by inhibiting podocyte apoptosis (21), reducing intraglomerular pressure by inducing afferent arteriolar vasoconstriction, and decreasing the proliferation of mesangial cells by blocking the cycle of mesangial cells (22). Voclosporin is a next-generation CNIs similar to cyclosporin. One of its functional groups is modified, allowing it to effectively combine with calcineurin and quickly remove metabolites without the need to detect drug concentrations (23). Although CNIs combined with hormone induction treatment of LN showed significant efficacy that was consistent with or better than that of CTX and MMF (24), some skepticism about its use remains, i.e., whether race and/or ethnicity influence response to CNIs as studies on CNIs are mainly conducted in Asia, whether the benefit of CNIs is largely nonimmunologic and associated with more flares, and whether long-term CNI exposure leads to worsening CKD in the LN population (3, 25). Larger CNI studies with more diverse patient populations, longer time horizons, and protocol biopsies are needed to help address these problems and clarify the evolving role of CNIs in the treatment of LN (26).

In recent years, there have been an increasing number of reports on the treatment of LN with biological agents, and more favorable outcomes have been achieved with less toxicity (27, 28). Compared with traditional therapeutic drugs, new biological agents target the pathogenesis of LN, offer rapid effects and few side effects, have gained increasing recognition, and have become a research hotspot (29). Of the eligible trials in this study, one-third of trials investigated the safety and efficacy of biologics in the treatment of LN. Biologics developed for LN can be largely categorized into B-cell directed therapies, T-cell directed agents, cytokine directed agents, and biologics that target complement components and signaling pathways. Biologics targeting B cells are generally aimed at depleting B cells, such as RTX, ocrelizumab, and obinutuzumab. These humanized monoclonal antibodies are directed against the B-cell surface marker CD20 and deplete CD20+ naive B cells and memory B cells, thereby eliminating sufficient B-cell precursors. Other biologics targeting B cells are anti-B lymphocyte stimulating factors that are generally aimed at blocking surface ligand-receptor interactions that promote B-cell activation and maturation, TLR expression, and antibody production (30). For example, belimumab and blisibimod target the B-cell ligand BAFF (B-cell activating factor), and atacicept, a fusion protein TACIIg (calcium modulator and cyclophilin ligand interactor), binds both B-cell ligands BAFF and APRIL (a proliferation-inducing ligand). Biologics directed at T cells focus on preventing T-cell costimulation, which is required for T-cell activation. For example, abatacept and RG2077 block interactions between costimulatory molecules CD80 and CD86 present on APCs with the costimulatory T-cell receptor CD28. BI 655064, iscalimab, and BG9588 are anti-CD40 or anti-CD40L antibodies that block CD154 binding to CD40. Itolizumab blocks the CD6 pathway. Milatuzumab blocks the CD74 pathway. In addition, biologics directed against cytokines, including TWEAK, TNF, IFN-γ, IL-6, IL-17, IL-23, and MIF, have also been investigated among these trials. These agents include BIIB023, infliximab, AMG 811, anifrolumab, etanercept, CNTO 136, secukinumab, guselkumab, and an anti-MIF antibody. In addition, other biologics targeting complement components, such as ravulizumab, APL-2, and narsoplimab, and the signaling pathway BMS-986165, which is a tyrosine kinase two signaling pathway inhibitor, have also been studied. Although biologics have shown promising success, to date, biologic agents for LN have yet to achieve the desired response and have considerable secondary effects. Thus, further and ongoing research is needed to determine safety, efficacy, optimal combination, dosage, and timing.

Stem cell therapy, which includes transplantation of hematopoietic stem cells (HSCs) and mesenchymal stem cells (MSCs), has become a new approach in the treatment of LN. HSCs can be obtained from bone marrow, umbilical cord blood, or peripheral blood. Mechanistically, agents are employed to remove the existing immune components in patients using a pretreatment regimen, such as CTX or anti-thymocyte globulin; then, HSCs are administered to rebuild the immune system (31). MSCs can be obtained from bone marrow (the nonhematopoietic component), umbilical cord, umbilical cord blood, adipose tissue, or embryonic tissue. Mechanisms have been proposed regarding the potential of MSCs to ameliorate LN, including correcting the immune imbalance, inducing immune tolerance and tissue repair, and improving organ function (32). Although some clinical studies have reported that stem cell transplantation (whether HSCs or MSCs) has a therapeutic effect on LN patients (33, 34), procedure-associated mortality is a concern. Further research is needed to optimize and improve these treatment options.

LN is more likely to occur in children (35), and childhood-onset LN exhibits a more active disease course and worse long-term survival than adult-onset LN (36, 37). However, recommendations for the management of childhood-onset LN published by American and European experts in pediatric SLE and LN are largely based on data extrapolated from studies in adults (38, 39). Compared with proliferative LN, pure MLN is associated with a lower risk of progression to end-stage renal disease (40). Therefore, the treatment of pure MLNs differs from that of proliferative LNs. However, insufficient evidence is available to recommend the best immunosuppressive treatment strategy for MLN at present given that well-powered clinical RCTs for MLN are lacking (41). According to this study, only 3.2% of trials included children, and 4.8% included patients with pure MLN. Once clinical remission has been achieved, the treatment of LN patients should change to maintenance therapy. The maintenance phase is important because despite clinical remission, histological activity is present in 44.4% of patients, and persistent kidney inflammation seems to increase the risk of renal relapse and chronic kidney disease progression (42). Among these trials, only 10.3% investigated maintenance therapy. Obviously, there is still a great shortage of research for MLN, childhood-onset LN, and maintenance treatment of LN, and we hope that more research institutions will conduct drug trials using these patients to establish optimal clinical treatment strategies.

In 2004, the International Committee of Medical Journal Editors (ICMJE) issued a publication policy requesting that prospective clinical trials need to be registered prior to patient inclusion (43). From this study, the number of clinical trial registrations for LN therapy has increased significantly since 2006. This increase may be related to this publication policy, as reported in other studies (44). The ICMJE believes that there is an ethical obligation to responsibly share data generated by interventional clinical trials because trial participants have put themselves at risk. In January 2016, the ICMJE published a proposal aimed at helping to create an environment in which the sharing of deidentified individual participant data becomes the norm. The ICMJE requires manuscripts submitted to ICMJE journals as of July 1, 2018 that report the results of clinical trials to include a data sharing statement, and clinical trials that begin enrolling participants on or after January 1, 2019 must include a data sharing plan in the trial's registration. Only 6.3% of the trials had IPD sharing statements in our study, but we believe that IPD sharing statements will be increasingly more common in future registered clinical trials. Approximately half (42.1%) of the trials in the study were completed; however, only 14.3% trials had results available on ClinicalTrials.gov, which is similar to that noted in other reports (45, 46). In summary, prospective clinical trials need to be registered prior to patient inclusion, must include IPD sharing statements in the trial's registration, and upload the results of completed studies to the Clinicatrials.gov registry in a timely fashion to make research more transparent, reduce publication bias, and ensure the safety of participants.

Our research still had some limitations: (1) This study only retrieved trials listed with ClinicalTrials.gov. Although approximately two-thirds of total global registrations and 375,253 research studies in all 50 states and 220 countries are listed on ClinicalTrials.gov, we might miss some trials registered in other registration centers. (2) All information pertaining to the conclusions reached in this study comes from ClinicalTrials.gov, and there are no other sources of information. The clinical trial registry may have changed and may not reflect the actual situation.

This study discussed the current situation and problems of clinical trials currently being conducted for LN on ClinicalTrials.gov. For researchers and physicians conducting future clinical trials for LN, some important points should be noted. (1) Large-scale, long-term, multicenter, and sufficient masked high-quality RCT trials with standardized inclusion/exclusion criteria and treatment effect evaluation systems should be conducted to generate good clinical evidence. (2) More energy and funding should be put into exploring biological products and stem cell therapies. (3) Trials for MLN, childhood-onset LN, and maintenance phase of LN are needed to establish optimal treatment strategies. (4) Prospective clinical trials need to be registered prior to inclusion in patients, must include IPD sharing statements in the trial's registration, and should update the latest results of the trial in a timely and complete manner to make the research more transparent, reduce publication bias, and ensure the safety of participants.

## Conclusions

The majority of clinical trials for LN therapy registered on ClinicalTrials.gov investigated the initial treatment of adult proliferative LN, and most of these trials were randomized, parallel assigned, and insufficiently masked interventional trials with small scale, short duration, various eligibility criteria, and outcome measures. We hope that more large-scale, long-term multicenter and high-quality RCT trials with standardized inclusion criteria/exclusion criteria and treatment effect evaluation systems will be conducted and that more energy and funding will be put into exploring biological products and stem cell therapies. In addition, trials for MLN, childhood-onset LN, and maintenance phase LN are needed to establish optimal treatment strategies.

## Data Availability Statement

The raw data supporting the conclusions of this article are included in the Supplementary Material. Further inquiries can be directed to the corresponding author.

## Author Contributions

XZ designed this study. YG and YW performed search, collected data, and performed analysis. YG wrote the manuscript. RL reviewed the manuscript. All authors contributed to the article and approved the submitted version.

## Conflict of Interest

The authors declare that the research was conducted in the absence of any commercial or financial relationships that could be construed as a potential conflict of interest.

## References

[1] Gergianaki I Fanouriakis A Repa A Tzanakakis M Adamichou C Pompieri A. Epidemiology and burden of systemic lupus erythematosus in a Southern European population: data from the community-based lupus registry of Crete, Greece. Ann Rheum Dis. (2017) 76:1992–2000. 10.1136/annrheumdis-2017-211206 28780511

[2] Hanly JG O'Keeffe AG Su L Urowitz MB Romero-Diaz J Gordon C. The frequency and outcome of lupus nephritis: results from an international inception cohort study. Rheumatology. (2016) 55:252–62. 10.1093/rheumatology/kev311 26342222 PMC4939728

[3] Mok CC Ying KY Yim CW Siu YP Tong KH To CH. Tacrolimus versus mycophenolate mofetil for induction therapy of lupus nephritis: a randomised controlled trial and long-term follow-up. Ann Rheum Dis. (2016) 75:30–6. 10.1136/annrheumdis-2014-206456 25550339

[4] Rathi M Goyal A Jaryal A Sharma A Gupta PK Ramachandran R. Comparison of low-dose intravenous cyclophosphamide with oral mycophenolate mofetil in the treatment of lupus nephritis. Kidney Int. (2016) 89:235–42. 10.1038/ki.2015.318 26489028

[5] Mok CC Ho LY Ying SKY Leung MC To CH Ng WL. Long-term outcome of a randomised controlled trial comparing tacrolimus with mycophenolate mofetil as induction therapy for active lupus nephritis. Ann Rheum Dis. (2020) 79:1070–6. 10.1136/annrheumdis-2020-217178 32448782

[6] Gasparotto M Gatto M Binda V Doria A Moroni G. Lupus nephritis: clinical presentations and outcomes in the 21st century. Rheumatology. (2020) 59(Suppl. 5):v39–51. 10.1093/rheumatology/keaa381 33280015 PMC7751166

[7] Tektonidou MG Dasgupta A Ward MM. Risk of end-stage renal disease in patients with lupus nephritis, 1971-2015: a systematic review and bayesian meta-analysis. Arthr Rheumatol. (2016) 68:1432–41. 10.1002/art.39594 26815601 PMC5071782

[8] Simes RJ. Publication bias: the case for an international registry of clinical trials. J Clin Oncol. (1986) 4:1529–41. 10.1200/JCO.1986.4.10.1529 3760920

[9] Pocock SJ Clayton TC Stone GW. Design of major randomized trials: part 3 of a 4-part series on statistics for clinical trials. J Am Coll Cardiol. (2015) 66:2757–66. 10.1016/j.jacc.2015.10.036 26700838

[10] Sessler DI Imrey PB. Clinical research methodology 3: randomized controlled trials. Anesth Analg. (2015) 121:1052–64. 10.1213/ANE.0000000000000862 26378705

[11] Schulz KF Grimes DA. Blinding in randomised trials: hiding who got what. Lancet. (2002) 359:696–700. 10.1016/S0140-6736(02)07816-9 11879884

[12] Psaty BM Prentice RL. Minimizing bias in randomized trials: the importance of blinding. JAMA. (2010) 304:793–4. 10.1001/jama.2010.1161 20716744

[13] Brophy JM. Multicenter trials, guidelines, and uncertainties - Do we know as much as we think we do? Int J Cardiol. (2015) 187:600–3. 10.1016/j.ijcard.2015.04.004 25863734

[14] Tamirou F D'Cruz D Sangle S Remy P Vasconcelos C Fiehn C. Long-term follow-up of the MAINTAIN nephritis trial, comparing azathioprine and mycophenolate mofetil as maintenance therapy of lupus nephritis. Ann Rheum Dis. (2016) 75:526–31. 10.1136/annrheumdis-2014-206897 25757867 PMC4789692

[15] Dall'Era M Cisternas MG Smilek DE Straub L Houssiau FA Cervera R. Predictors of long-term renal outcome in lupus nephritis trials: lessons learned from the Euro-Lupus Nephritis cohort. Arthr Rheumatol. (2015) 67:1305–13. 10.1002/art.39026 25605554

[16] Mackay M Dall'Era M Fishbein J Kalunian K Lesser M Sanchez-Guerrero J. Establishing surrogate kidney end points for lupus nephritis clinical trials: development and validation of a novel approach to predict future kidney outcomes. Arthr Rheumatol. (2019) 71:411–9. 10.1002/art.40724 30225865

[17] Hahn BH McMahon MA Wilkinson A Wallace WD Daikh DI Fitzgerald JD. American college of rheumatology guidelines for screening, treatment, and management of lupus nephritis. Arthritis Care Res. (2012) 64:797–808. 10.1002/acr.21664 22556106 PMC3437757

[18] Fanouriakis A Kostopoulou M Cheema K Anders HJ Aringer M Bajema I. 2019 update of the joint European league against rheumatism and European renal association-European dialysis and transplant association (EULAR/ERA-EDTA) recommendations for the management of lupus nephritis. Ann Rheum Dis. (2020) 79:713–23. 10.1136/annrheumdis-2020-216924 32220834

[19] Chinese lupus nephritis diagnosis and treatment guidelines compilation group. Guidelines for the diagnosis and treatment of lupus nephritis in China. Nat Med J China. (2019) 99:3441–55. 10.3760/cma.j.issn.0376-2491.2019.44.001

[20] Mok CC. Calcineurin inhibitors in systemic lupus erythematosus. Best Pract Res Clin Rheumatol. (2017) 31:429–38. 10.1016/j.berh.2017.09.010 29224682

[21] Liao R Liu Q Zheng Z Fan J Peng W Kong Q. Tacrolimus protects podocytes from injury in lupus nephritis partly by stabilizing the cytoskeleton and inhibiting podocyte apoptosis. PLoS ONE. (2015) 10:e0132724. 10.1371/journal.pone.0132724 26161538 PMC4498640

[22] Gao Y Yang H Wang Y Tian J Li R Zhou X. Evaluation of the inhibitory effect of tacrolimus combined with mycophenolate mofetil on mesangial cell proliferation based on the cell cycle. Int J Mol Med. (2020) 46:1582–92. 10.3892/ijmm.2020.4696 32945359 PMC7447332

[23] Rovin BH Solomons N Pendergraft WF 3rd Dooley MA Tumlin J Romero-Diaz J. A randomized, controlled double-blind study comparing the efficacy and safety of dose-ranging voclosporin with placebo in achieving remission in patients with active lupus nephritis. Kidney Int. (2019) 95:219–31. 10.1016/j.kint.2018.08.025 30420324

[24] Cai J Liu XY. Chinese expert consensus on tacrolimus in lupus nephritis. Chin J Rheumatol. (2017) 21:483–5. 10.3760/cma.j.issn.1007-7480.2017.07.012

[25] Fernandez Nieto M Jayne DR. Con: the use of calcineurin inhibitors in the treatment of lupus nephritis. Nephrol Dial Transplant. (2016) 31:1567–71. 10.1093/ndt/gfw291 27591328

[26] Peleg Y Bomback AS Radhakrishnan J. The evolving role of calcineurin inhibitors in treating lupus nephritis. Clin J Am Soc Nephrol. (2020) 15:1066–72. 10.2215/CJN.13761119 32152065 PMC7341791

[27] Sciascia S Radin M Yazdany J Levy RA Roccatello D Dall'Era M. Efficacy of belimumab on renal outcomes in patients with systemic lupus erythematosus: a systematic review. Autoimmun Rev. (2017) 16:287–93. 10.1016/j.autrev.2017.01.010 28147262

[28] Zhong Z Li H Zhong H Zhou T. Clinical efficacy and safety of rituximab in lupus nephritis. Drug Design Dev Ther. (2019) 13:845–56. 10.2147/DDDT.S195113 30880917 PMC6417005

[29] Hobeika L Ng L Lee IJ. Moving forward with biologics in lupus nephritis. Adv Chronic Kidney Dis. (2019) 26:338–50. 10.1053/j.ackd.2019.08.008 31733718

[30] Tsokos GC Lo MS Costa Reis P Sullivan KE. New insights into the immunopathogenesis of systemic lupus erythematosus. Nat Rev Rheumatol. (2016) 12:716–30. 10.1038/nrrheum.2016.186 27872476

[31] Snowden JA Saccardi R Allez M Ardizzone S Arnold R Cervera R. Haematopoietic SCT in severe autoimmune diseases: updated guidelines of the European Group for blood and marrow transplantation. Bone Marrow Trans. (2012) 47:770–90. 10.1038/bmt.2011.185 22002489 PMC3371413

[32] Sattwika PD Mustafa R Paramaiswari A Herningtyas EH. Stem cells for lupus nephritis: a concise review of current knowledge. Lupus. (2018) 27:1881–97. 10.1177/0961203318793206 30099942

[33] Deng D Zhang P Guo Y Lim TO. A randomised double-blind, placebo-controlled trial of allogeneic umbilical cord-derived mesenchymal stem cell for lupus nephritis. Ann Rheum Dis. (2017) 76:1436–9. 10.1136/annrheumdis-2017-211073 28478399

[34] Huang X Chen W Ren G Zhao L Guo J Gong D. Autologous hematopoietic stem cell transplantation for refractory lupus nephritis. Clin J Am Soc Nephrol. (2019) 14:719–27. 10.2215/CJN.10570918 30979713 PMC6500938

[35] Sassi RH Hendler JV Piccoli GF Gasparin AA da Silva Chakr RM Brenol JC. Age of onset influences on clinical and laboratory profile of patients with systemic lupus erythematosus. Clin Rheumatol. (2017) 36:89–95. 10.1007/s10067-016-3478-4 27858177

[36] Tektonidou MG Lewandowski LB Hu J Dasgupta A Ward MM. Survival in adults and children with systemic lupus erythematosus: a systematic review and bayesian meta-analysis of studies from 1950 to 2016. Ann Rheum Dis. (2017) 76:2009–16. 10.1136/annrheumdis-2017-211663 28794077

[37] Oni L Wright RD Marks S Beresford MW Tullus K. Kidney outcomes for children with lupus nephritis. Pediatr Nephrol. (2020) 36:1377–85. 10.1007/s00467-020-04686-1 32725543 PMC8084759

[38] Groot N de Graeff N Avcin T Bader-Meunier B Brogan P Dolezalova P. European evidence-based recommendations for diagnosis and treatment of childhood-onset systemic lupus erythematosus: the SHARE initiative. Ann Rheum Dis. (2017) 76:1788–96. 10.1136/annrheumdis-2016-210960 28630236

[39] Mina R von Scheven E Ardoin SP Eberhard BA Punaro M Ilowite N. Consensus treatment plans for induction therapy of newly diagnosed proliferative lupus nephritis in juvenile systemic lupus erythematosus. Arthritis Care Res. (2012) 64:375–83. 10.1002/acr.21558 22162255 PMC3457803

[40] Mok CC. Towards new avenues in the management of lupus glomerulonephritis. Nat Rev Rheumatol. (2016) 12:221–34. 10.1038/nrrheum.2015.174 26729459

[41] Almaani S Parikh SV. Membranous lupus nephritis: a clinical review. Adv Chronic Kidney Dis. (2019) 26:393–403. 10.1053/j.ackd.2019.08.009 31733724

[42] Parikh SV Nagaraja HN Hebert L Rovin BH. Renal flare as a predictor of incident and progressive CKD in patients with lupus nephritis. Clin J Am Soc Nephrol. (2014) 9:279–84. 10.2215/CJN.05040513 24262502 PMC3913239

[43] De Angelis C Drazen JM Frizelle FA Haug C Hoey J Horton R. Clinical trial registration: a statement from the international committee of medical journal editors. Lancet. (2004) 364:911–2. 10.1016/S0140-6736(04)17034-7 15364170

[44] Wang YB Lv G Xu FH Ma LL Yao YM. Comprehensive survey of clinical trials registration for melanoma immunotherapy in the ClinicalTrials.gov. Front Pharmacol. (2019) 10:1539. 10.3389/fphar.2019.01539 31998135 PMC6966167

[45] Ma LL Qiu Y Song MN Chen Y Qu JX Li BH. Clinical trial registration and reporting: drug therapy and prevention of cardiac-related infections. Front Pharmacol. (2019) 10:757. 10.3389/fphar.2019.00757 31333470 PMC6624234

[46] Chen L Su Y Quan L Zhang Y Du L. Clinical trials focusing on drug control and prevention of ventilator-associated pneumonia: a comprehensive analysis of trials registered on ClinicalTrials.gov. Front Pharmacol. (2018) 9:1574. 10.3389/fphar.2018.01574 30863312 PMC6399618

